# Hydrogel Based on Chitosan/Gelatin/Poly(Vinyl Alcohol) for In Vitro Human Auricular Chondrocyte Culture

**DOI:** 10.3390/polym16040479

**Published:** 2024-02-08

**Authors:** Carmina Ortega-Sánchez, Yaaziel Melgarejo-Ramírez, Rogelio Rodríguez-Rodríguez, Jorge Armando Jiménez-Ávalos, David M. Giraldo-Gomez, Claudia Gutiérrez-Gómez, Jacobo Rodriguez-Campos, Gabriel Luna-Bárcenas, Cristina Velasquillo, Valentín Martínez-López, Zaira Y. García-Carvajal

**Affiliations:** 1 Laboratorio de Biotecnología, Unidad de Gerociencias, Instituto Nacional de Rehabilitación Luis Guillermo Ibarra Ibarra, Ciudad de México 14389, Mexico; carminaortega52@gmail.com (C.O.-S.); yaazielmr@gmail.com (Y.M.-R.); 2 Biotecnología Médica y Farmacéutica, Centro de Investigación y Asistencia en Tecnología y Diseño del Estado de Jalisco A.C. (CIATEJ), Av. Normalistas No. 800, Col. Colinas de la Normal, Guadalajara 44270, Jalisco, Mexico; rogelio.rodriguez4085@academicos.udg.mx (R.R.-R.); avalos.joar@gmail.com (J.A.J.-Á.); 3 Unidad de Microscopia, Departamento de Biología Celular y Tisular, Facultad de Medicina, Universidad Nacional Autónoma de México, Avenida Universidad 3000, Circuito Interior, Edificio “A” Planta Baja, Ciudad Universitaria, Coyoacán, Ciudad de México 04510, Mexico; davidgiraldo@comunidad.unam.mx; 4 División de Cirugía Plástica y Reconstructiva, Hospital General Dr. Manuel Gea González, Ciudad de México 14080, Mexico; dra.claugg8@gmail.com; 5 Servicios Analíticos y Metrológicos, Centro de Investigación y Asistencia en Tecnología y Diseño del Estado de Jalisco A.C. (CIATEJ), Av. Normalistas No. 800, Col. Colinas de la Normal, Guadalajara 44270, Jalisco, Mexico; jarodriguez@ciatej.mx; 6 Institute of Advanced Materials for Sustainable Manufacturing Tecnológico de Monterrey, Epigmenio González 500, San Pablo, Santiago de Querétaro 76130, Querétaro, Mexico; gabriel.luna@cinvestav.mx; 7 Unidad de Ingeniería de Tejidos Terapia Celular y Medicina Regenerativa, Instituto Nacional de Rehabilitación Luis Guillermo Ibarra Ibarra, Ciudad de México 14389, Mexico

**Keywords:** composite hydrogel, three-dimensional hydrogel, chitosan, gelatin, tissue engineering, auricular chondrocytes culture

## Abstract

Three-dimensional (3D) hydrogels provide tissue-like complexities and allow for the spatial orientation of cells, leading to more realistic cellular responses in pathophysiological environments. There is a growing interest in developing multifunctional hydrogels using ternary mixtures for biomedical applications. This study examined the biocompatibility and suitability of human auricular chondrocytes from microtia cultured onto steam-sterilized 3D Chitosan/Gelatin/Poly(Vinyl Alcohol) (CS/Gel/PVA) hydrogels as scaffolds for tissue engineering applications. Hydrogels were prepared in a polymer ratio (1:1:1) through freezing/thawing and freeze-drying and were sterilized by autoclaving. The macrostructure of the resulting hydrogels was investigated by scanning electron microscopy (SEM), showing a heterogeneous macroporous structure with a pore size between 50 and 500 μm. Fourier-transform infrared (FTIR) spectra showed that the three polymers interacted through hydrogen bonding between the amino and hydroxyl moieties. The profile of amino acids present in the gelatin and the hydrogel was determined by ultra-performance liquid chromatography (UPLC), suggesting that the majority of amino acids interacted during the formation of the hydrogel. The cytocompatibility, viability, cell growth and formation of extracellular matrix (ECM) proteins were evaluated to demonstrate the suitability and functionality of the 3D hydrogels for the culture of auricular chondrocytes. The cytocompatibility of the 3D hydrogels was confirmed using a 3-(4,5-dimethylthiazol-2-yl)-2,5-diphenyltetrazolium bromide (MTT) assay, reaching 100% viability after 72 h. Chondrocyte viability showed a high affinity of chondrocytes for the hydrogel after 14 days, using the Live/Dead assay. The chondrocyte attachment onto the 3D hydrogels and the formation of an ECM were observed using SEM. Immunofluorescence confirmed the expression of elastin, aggrecan and type II collagen, three of the main components found in an elastic cartilage extracellular matrix. These results demonstrate the suitability and functionality of a CS/Gel/PVA hydrogel as a 3D support for the auricular chondrocytes culture, suggesting that these hydrogels are a potential biomaterial for cartilage tissue engineering applications, aimed at the regeneration of elastic cartilage.

## 1. Introduction

Hydrogels are polymeric materials with a hydrophilic structure that allows for the storage of large amounts of water and biological fluids [1,2,3,4,5,6,7,8], and are ideal for in vitro cell cultures, as they provide a supportive matrix that mimics a natural microenvironment [1,9,10]. Three-dimensional (3D) hydrogels provide tissue-like complexities and allow for the spatial orientation of cells, leading to more realistic cellular responses in pathophysiological environments [7,10,11]. Cell behavior in 3D hydrogels is markedly variable, not only between populations of different types of cells, but also between different tissues [5,6,7,8,9,10,11,12]. The cellular microenvironment plays a vital role in cell functions, from controlling morphology to activating a wide range of factors regulating cell growth, proliferation, differentiation and migration [10,11,12].

Chitosan (CS) is a deacetylated linear polysaccharide, derivative of chitin and composed of variable amounts of attached residues (β1→4) of N-acetyl-2 amino-2-deoxy-D-glucose (glucosamine, GlcN) and 2-amino-residues of 2-deoxy-D-glucose (N-acetyl-glucosamine, GlcNAc) [13,14]. Gelatin (Gel) is a partially hydrolyzed collagen that promotes cell adhesion, proliferation and migration and is also used as a gelling agent carrier for encapsulating bioactive molecules [15,16]. It is mechanically weak and requires chemical modification or combination with other materials to form a hydrogel [17]. Poly(vinyl alcohol) (PVA) is a biodegradable hydrophilic synthetic polymer with excellent biocompatibility [18]. There is a growing interest in developing multifunctional hydrogels [4] using ternary mixtures for biomedical applications [19]. We have previously reported that human adipose-derived mesenchymal stromal cells (AD-hMSCs) displayed chondroinductive properties after being cultured onto bidimensional CS/Gel/PVA hydrogels and following their exposure to chondrogenic stimulation media [20]. The synthesis of 3D hydrogels resulted in open and interconnected macroporous structures, allowing gas and nutrient exchange and improving their mechanical properties. Furthermore, 3D CS/Gel/PVA hydrogels were non-toxic to HT29 cells [21,22] and BRIN-BD11 cells [23], demonstrating their potential use as scaffolds for tissue engineering applications.

Advances in material sciences have the potential to benefit tissue engineering techniques aimed at the treatment of congenital deformities within the field of plastic and reconstructive surgery [24,25,26]. Three-dimensional hydrogels are used to support the growth of chondrocytes, the cartilage cells, offering an optimal microenvironment similar to native elastic cartilage [25,27,28,29,30,31,32,33]. They can positively affect cell morphology, proliferation and differentiation of auricular chondrocytes [32,34,35,36,37,38,39]. This study aimed to examine the biocompatibility and suitability of human auricular chondrocytes from microtia cultured onto steam-sterilized 3D CS/Gel/PVA hydrogels as scaffolds for tissue engineering applications.

## 2. Materials and Methods

Ethical Considerations

This study was approved by the Institutional Research Committee and Ethics Committee (Register number: INRLGII 85/19). Pediatric patients were recruited from the microtia clinic at the National Institute of Rehabilitation Luis Guillermo Ibarra Ibarra and from the General Hospital Dr. Manuel Gea González in Mexico City. After informed consent was granted, the microtia cartilage remaining from costal cartilage graft ear reconstruction surgeries was donated.

Chemicals

Low molecular weight chitosan (CS, deacetylation degree ~92.2%), type B gelatin from bovine skin (Gel, Bloom ~75), poly(vinyl alcohol) (PVA, molecular weight ~89 kDa and hydrolysis degree ~99.8%), o-xylene and reagents for the cytotoxicity assay were supplied by Sigma Aldrich (St. Louis, MO, USA). Solvents and reagents were analytical grade.

### 2.1. Chitosan/Gelatin/Poly(Vinyl Alcohol) Hydrogel Preparation and Sterilization

#### 2.1.1. Ternary Polymer Solutions

CS solution (2.5 wt %) was obtained by dissolving chitosan powder in 0.4 M acetic acid solution and gently stirring it for 12 h at 25 °C. Gel solution (2.5 wt %) was prepared by dissolving gelatin powder in distilled water and gently stirring it for 2 h at 37 °C. PVA solution (2.5 wt %) was prepared by dissolving the powder in distilled water and gently stirring it for 2 h at 80 °C.

#### 2.1.2. Sterilized Hydrogel Preparation

CS/Gel/PVS hydrogels were fabricated by blending the polymer solutions in a 1:1:1 ratio, according to the method previously described by Rodríguez-Rodríguez [21]. The preparation process consisted of four steps: (1) a freeze–thaw cycle, (2) a lyophilization process, (3) chemical treatment with o-xylene as a porogen template, and (4) steam sterilization. The pH of the ternary solutions was adjusted to 4.0, to obtain homogeneous solutions. After that, the polymeric solution was poured into a plastic mold (14 mL) and frozen at −80 °C for 24 h with a subsequent freeze-drying process using a freeze dryer (Telstar LyoQuest, Terrassa, Spain) and a vacuum of 0.2 mbar for 72 h. Dried samples were neutralized using sodium hydroxide solution (NaOH, 0.1 N) for 30 min. Afterwards, to induce porosity, hydrogels were put into the o-xylene solution for 15 min under mechanical agitation at 200 rpm, using an orbital shaker incubator at 37 °C (Luzeren, Hangzhou, China). The CS/Gel/PVA hydrogels were washed with ethyl alcohol from 100 to 0% *v*/*v* to remove residual xylene. Finally, hydrogels were sterilized in phosphate-buffered saline (PBS) using a steam autoclave SE 510 (Yamato Scientific, Tokyo, Japan) at 121 °C and 103.4 KPa for 15 min [21].

### 2.2. Characterization of Chitosan/Gelatin/Poly(Vinyl Alcohol) Hydrogels

#### 2.2.1. Morphological Analysis

Scanning electron microscopy (SEM) analysis was performed to confirm the morphological properties of the steam-sterilized CS/Gel/PVA hydrogels. Freeze-dried hydrogels were mounted on carbon stubs with double-sided adhesive tape without coating. In addition, energy-dispersive X-ray spectrometer analysis (EDS, JSM-7100f, JEOL, Tokyo, Japan) was performed, to determine the elemental component of the hydrogel.

#### 2.2.2. Fourier-Transform Infrared Spectroscopy

Fourier-transform infrared–attenuated total reflectance (FTIR–ATR) was used to identify the structural properties of the CS/Gel/PVA hydrogels. Freeze-dried hydrogels were lyophilized, ground with a grinder, and placed on the ATR surface of the Spectrum GX system (Perkin Elmer, Branford, CT, USA). Spectra were obtained with a resolution of 1 cm^−1^ over 24 scans, ranging from 4000 to 650 cm^−1^.

#### 2.2.3. Amino Acid Profile

The amino acid profiles of the type B gelatin from bovine skin and the CS/Gel/PVA hydrogels were determined by ultra-performance liquid chromatography (UPLC). Ten milligrams of the sample were hydrolyzed for 24 h with 0.25 N HCl at 110 °C. The hydrolysate was filtered (Whatman No. 2) and dried at 60 °C in a rotary evaporator. The dry hydrolysate was resuspended in 1 mL 0.1 N HCl, and the solution was filtered through a 0.22 µm membrane (AOAC, 2005; Method 982.30). The standards and hydrolysate (10 µL) were derivatized with AccQ-Fluor borate buffer (70 µL) and AccQ-Fluor reagent (20 µL) from Waters. The derivatized was separated and quantified by UPLC Acquity class H with an Acquity diode array detector (Waters Corporation, Milford, MA, USA) and AccQ-Tag ultra C18 column (2.1 x 100 mm). A particle size of 1.7 µm was used in this study. The separation was performed using the following two solvents: (A) AccQ-Tag ultra A/water in proportion 5:95 (*v*/*v*) and (B) AccQ-Tag ultra B (Waters^®^). The gradient elution used was as follows: holding at 0.1% B for 0 to 0.54 min, then 0.1% to 9.1% (B) from 0.54 to 5.74 min, 9.1% to 21.2% (B) from 5.74 to 7.74 min and 21.2% to 59.6% from 7.74 to 8.04. The injection volume was 10 μL, the column temperature was 55 °C and the analysis time was 10 min. Detection was carried out at 260 nm. Data acquisition was performed with Empower 3 system software (WatersCorporation, Milford, MA, USA). Amino acid quantification was carried out using external standards for each amino acid (WatersCorporation, Milford, MA, USA) [40].

### 2.3. Culture of Chondrocytes in Chitosan/Gelatin/Poly(Vinyl Alcohol) Hydrogels

#### 2.3.1. Isolation and Culture of Human Elastic Chondrocytes

Elastic cartilage remnants were donated from 7- to 10-year-old patients with unilateral congenital microtia who underwent autologous costal cartilage reconstruction. Briefly, auricular remnants were transported in 10% antibiotic–antimycotic/DMEM-F12 media (Gibco^®^, U.S., New York, NY, USA) under sterile conditions. Tissue remnants were minced and enzymatically predigested with 0.25% trypsin–EDTA (Gibco^®^, U.S., New York, NY, USA) for 30 min. Afterward, digestion with type II collagenase (3 mg/mL, Worthington Biochemical Corporation), was performed for 5 h at 37 °C under constant agitation. Cell viability was determined using trypan blue. Chondrocytes were seeded onto CS/Gel/PVA hydrogels at a cell density of 2 × 10^4^ cells/cm^2^ and kept in DMEM-F12/10% FBS/1% antibiotic–antimycotic supplemented media (Gibco^®^, U.S., New York, NY, USA) until confluence. At this point, the cell-laden hydrogels were called constructs. Chondrocyte isolation and culture were conducted under standard conditions at 37 °C, 5% CO_2_, and 95% relative humidity.

#### 2.3.2. Cytotoxicity Assay

In vitro chondrocyte cytocompatibility with the steam-sterilized CS/Gel/PVA hydrogels was evaluated using supernatant dilutions (extracts), according to ISO 10993-5 [41]. Testing was performed using the (3-(4,5-dimethyl thiazolyl-2) 2,5-diphenyltetrazolium bromide (MTT, Thermo Fisher Scientific, Hillsboro, OR, USA) assay. Sterile hydrogels (~500 mg) were statically incubated in 5 mL of supplemented media (DMEM-F12/10% FBS/1% antibiotic–antimycotic) at 37 °C for 72 h. After this, extracts were diluted in supplemented media to obtain each working concentration tested: 100, 50, 25, 12.5, and 0% *v*/*v*.

Auricular chondrocytes were seeded in 96-well plates at a density of 5 × 10^3^ cells per well and cultured for 24 h. Afterwards, cell culture media in each well were replaced with an equal volume of the different hydrogel extract dilutions and incubated for 24 and 72 h in a 5% CO_2_ humidified atmosphere. Chondrocytes exposed to 1.0% *v*/*v* sodium dodecyl sulfate (SDS, Sigma-Aldrich) were used as positive cytotoxic controls for the same time points. At every time point, extract dilutions were removed from the wells, replaced with 100 µL of MTT solution (0.5 mg/mL) and incubated at 37 °C for 4 h. Formazan crystals were dissolved with isopropanol/dimethyl sulfoxide (DMSO) solution and transferred to a new 96-well plate. Absorbance was measured using a Multiskan GO spectrophotometer (Thermo Scientific, Pittsburgh, PA, USA) at 570 nm, and cell viability was calculated with the following formula:

$$\%\;cell\;viability=\frac{Is}{Ic}\times 100$$

where *Is* corresponds to the absorbance of the cells exposed to the extract, and *Ic* is the absorbance of the non-exposed cells.

#### 2.3.3. Cell Viability Assay

Chondrocyte viability within the CS/Gel/PVA hydrogel constructs was evaluated with a modified protocol from the LIVE/DEAD^®^ viability/cytotoxicity kit (Molecular Probes™ Invitrogen, Carlsbad, CA, USA) [42] after 7 and 14 days. Briefly, 5 × 10^5^ human elastic chondrocytes (third passage) were resuspended in supplemented media (80 µL), injected into 8 mm diameter hydrogel discs using an insulin syringe (U-100 Becton Dickinson, Mexico City, Mexico) and incubated for 1 h at 37 °C and 5% CO_2_. After that, constructs were entirely covered with supplemented media (Gibco^®^, U.S., New York, NY, USA) with periodic media changes every 2–3 days for 7 and 14 days. Assays were performed in triplicate at each time point. Constructs were washed three times with PBS and incubated in Hanks balanced salt solution (HBSS) containing 4 mM calcein-AM and 1 mM ethidium homodimer (EthD-1) for 45 min at 37 °C and 5% CO_2_. Then, constructs were examined with an LSM 780 confocal fluorescence microscope (Carl Zeiss, Oberkochen, Baden-Württemberg, Germany). Cell viability was determined using ImageJ software (version 1.52a, Wayne Rasband, National Institutes of Health, Bethesda, MD, USA).

#### 2.3.4. Cell Attachment

Chondrocyte attachment onto CS/Gel/PVA hydrogels was visualized using scanning electron microscopy (SEM) (16538, Electron Microscopy Science, Hatfield, PA, USA). Constructs (5 × 10^5^ cells) were fixed with 2.5% *v*/*v* glutaraldehyde (Sigma-Aldrich) in 0.1 M sodium cacodylate (Sigma-Aldrich) at pH 7.2 for 24 h. Samples were washed for 5 min in 0.1 M PBS at pH 7.4 and further dehydrated for 10 min in a serially diluted ethanol solution, ranging from 30% to 99.99%, for 30 min each wash (E7148, Sigma-Aldrich, Merck, Darmstadt, Germany). All specimens were dried with a critical point dryer CO_2_ chamber (K850, Quorum Technologies, Kent, UK). Images were acquired with a FIB-SEM Crossbeam 550 field emission microscope (Carl Zeiss, Oberkochen, Baden-Württemberg, Germany) at 5 kV.

#### 2.3.5. Immunofluorescence

After 7 and 14 days in culture, constructs (5 × 10^5^ cells) were fixed with 2% *v*/*v* paraformaldehyde (Sigma-Aldrich) for 20 min and included in Tissue-Tek^®^. Membrane permeabilization was carried out by incubating constructs in 0.1% Triton X-100 in PBS for 1 h at room temperature. Then, blocking was undertaken with 1% bovine serum albumin/PBS for 2 h. Constructs were incubated overnight with primary antibodies against type II collagen (ab34712, 1:100), Sox9 (ab3697, 1:100), aggrecan (ab36861, 1:100), elastin in monolayer (ab21610 1:100) and elastin in constructs (ab9519, 1:100), type I collagen (ab6308, 1:100) and Runx2 (ab76956, 1:100) at 4 °C. The secondary antibodies, anti-Mouse IgG (H+L) (1:300), FITC conjugated (Jackson Immunoresearch, #115–095–003) and goat anti-rabbit, Alexa Fluor 488 (Ab150073) or Alexa Fluor 594 (Ab150120), were incubated for 2 h at 37 °C. Finally, nuclear counterstaining was undertaken with 4′,6-diamidino-2-phenylindole (DAPI). Negative controls were not labeled with primary antibodies. Slides were mounted with fluoroshield™ (Cat: F6937-2ML), and images were taken with an Axio Observer inverted microscope A1 (Carl Zeiss, Oberkochen, Baden-Württemberg, Germany). Autofluorescence was reduced to allow for positive signal identification via confocal microscopy LSM 780 (Carl Zeiss).

**Statistical analysis**

Data were presented as three independent experiments’ mean ± standard deviations (SD) (n = 3). Significant differences were considered to be a *p*-value of < 0.05. A two-way ANOVA, followed by Tukey’s test, a multiple comparisons test and a Bonferroni’s comparison test, were performed using GraphPad Prism version 9.4.1 for macOS and GraphPad software (San Diego, CA, USA).

## 3. Results and Discussion

This study aimed to examine the suitability of sterilized CS/Gel/PVA hydrogels as scaffolds for culturing human auricular chondrocytes from microtia patients, in an environment acting as a scaffold, while maintaining the elastic chondrocyte phenotype for auricular tissue engineering applications.

CS/Gel/PVA-based hydrogels have proven to be a versatile platform for supporting various cell types for applications in tissue engineering and wound healing due to their natural bioactivity, biocompatibility, biodegradability, cellular adaptability and cell behavior [20,21,23,43].

Our results show the importance of generating a support biomaterial under controlled conditions with topological and architectural features to improve a 3D micro ambient that favors cell adhesion and interaction for supporting cell growth, for cartilage tissue engineering strategies.

### 3.1. Chitosan/Gelatin/Poly(Vinyl Alcohol) Hydrogel Preparation

**Figure 1:**
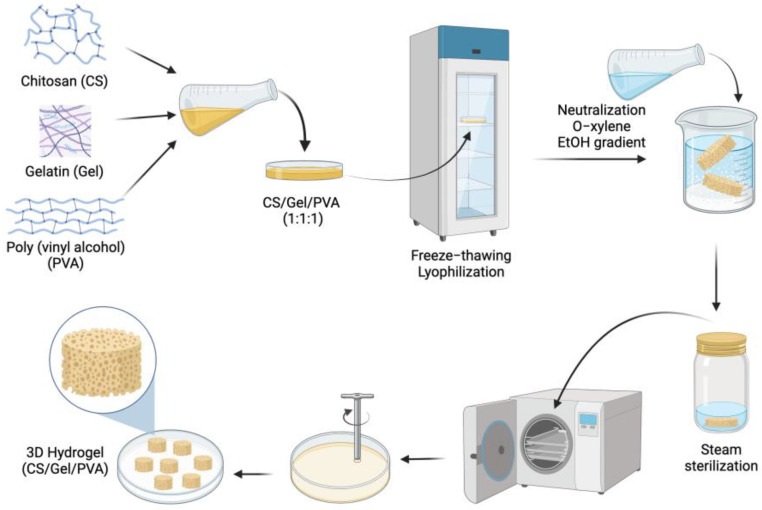
Schematic process of preparing the CS/Gel/PVA hydrogel.

The hydrogels were characterized by their macrostructure, chemical interactions, amino acid profile and ability to promote the growth of auricular chondrocytes in a 3D microenvironment. The CS/Gel/PVA ternary blend was used as part of the formulation hydrogel due to its biocompatibility [20,21,23,43,44,45] and biodegradability [23,46]. The process followed for preparing hydrogels is outlined in Figure 1. The samples were physically crosslinked by the freeze–thaw/lyophilization process and immersed in NaOH to promote the physical gelation of the deprotonated amino groups of the CS (pKa = 6.3–7.0) [21,38]. The o-xylene induced porosity in the hydrogels, due to its performance as a porogenic template [21,47].

This manufacturing process is feasible, robust, and easily reproducible, providing a reliable method to produce hydrogels with customized shapes and maintaining a pattern in the pore formation [21,46], which is particularly important for cell seeding [47]. The shape of hydrogels for auricular cartilage repair should have sufficient elasticity, flexibility, mechanical strength, and physical stability to support auricular chondrocyte growth [27,48,49].

### 3.2. Characterization of Chitosan/Gelatin/Poly(Vinyl Alcohol) Hydrogels

#### 3.2.1. Morphological Analysis

**Figure 2:**
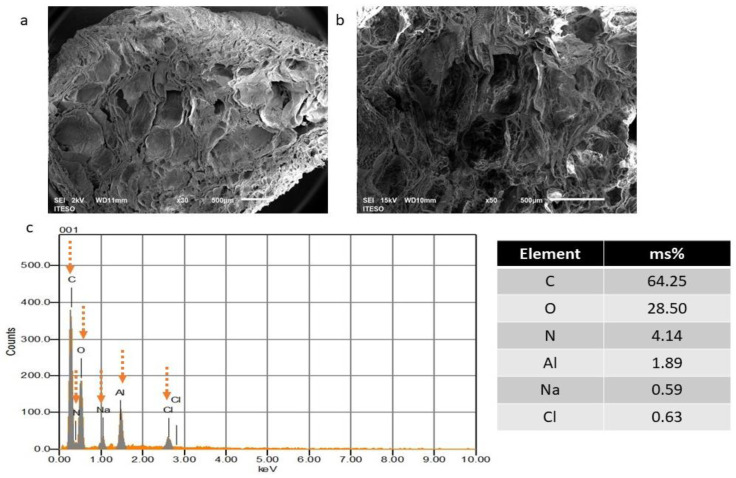
Morphological analysis of CS/Gel/PVA hydrogels. Scanning electron microscope (SEM) images showed heterogeneous channels with pore sizes ranging between 50 and 500 μm. Image magnification: 30× (**a**) and 50× (**b**). Scale bars are 500 µm. Energy-dispersive X-ray spectrometer (EDS) analysis (**c**). Dotted arrows indicate sharp peaks for each element.

The morphological characteristics of the hydrogels were observed. The SEM images revealed that the hydrogels possess a tridimensional (3D) structure with several irregular macroporous, open and closed channels with heterogeneous pore sizes and diameters ranging from 50 to 1500 μm (Figure 2a,b). More elongated and larger pores are present in some cross-sections. These porous structures and the heterogeneity in the size dimensions permit the adequate transport of nutrients, gases and molecules to ensure cell survival and cell functions inside the 3D hydrogel [21]. In our previous research, a different batch of hydrogels was prepared, following the same manufacturing process. Despite the heterogeneity in forming the 3D network and pore dimensions, the manufacturing process allowed control over the formation pattern of interconnected and porous channels, demonstrating the reproducibility of the manufacturing process [21].

Sánchez-Cardona et al. (2021) prepared a hydrogel similar to our research, in terms of their polymer blend proportions of CS/Gel/PVA (1:1:1 *w*/*w*) and polymer concentrations (2 wt %). The process preparation consisted of a freeze–thawing (nine cycles at −50 °C for 8 h and 25 °C for 8 h) with a subsequent lyophilization process without neutralization and xylene treatments. Under these conditions, the hydrogels showed homogeneity in the macroporous structure of the hydrogels, with small pore sizes (pore diameter sizes between 0.6 and 265 µm), but the preparation time increased significantly [23].

In addition, energy dispersive X-ray spectroscopy (EDX) analysis was conducted to analyze the elemental composition of the CS/Gel/PVA hydrogels. The EDX spectrum (Figure 2c) reveals sharp peaks of C (64.25%), O (28.50%) and N (4.14%), which can be attributed to the natural polymers (CS and Gel) and PVA (C and O) [50]. The peaks of Cl (0.63%) and Na (0.59%) are due to the PBS (mainly composed of sodium chloride) remaining in the hydrogels after the sterilization process [21]. The Al peak was detected, presumably due to the aluminum foil used to cover and store samples until SEM analysis.

#### 3.2.2. Fourier-Transform Infrared Spectroscopy

**Figure 3:**
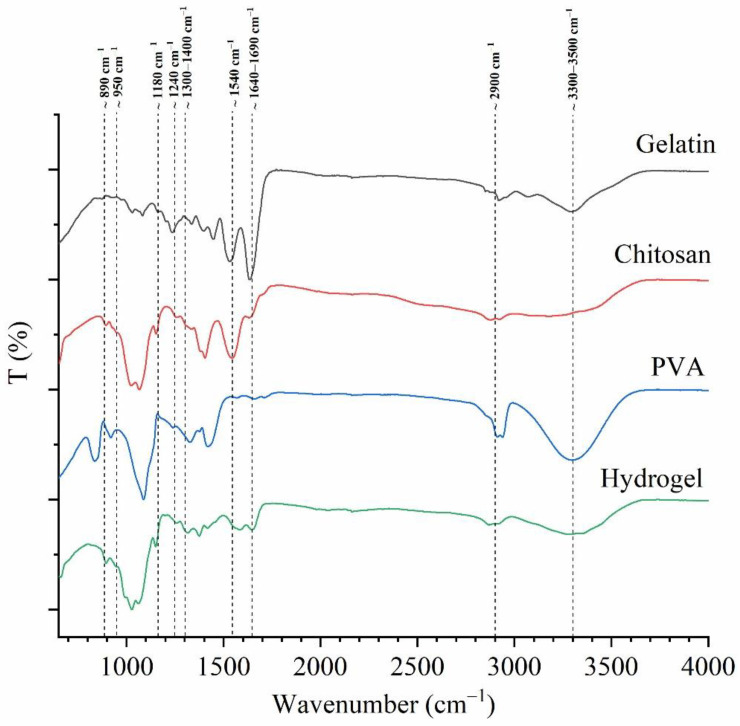
ATR–FTIR spectra of pristine Gel, CS and PVA and of dried CS/Gel/PVA hydrogel. The transmittance is shown on the left axis.

The results of the FT-IR spectroscopy were analyzed to understand the chemical interactions between the polymers and to confirm the absence of any unfavorable interactions with the o-xylene treatment of the hydrogels. The spectra of the pristine polymers (CS, Gel, and PVA) were used as controls and compared with the hydrogels (Figure 3). The FTIR spectra were described between 2000 and 600 cm^−1^ peak regions. The characteristic bands (peaks) of pristine polymers are shown in Table 1. The IR spectrum of pristine CS (Figure 3) showed peaks around 890–900 cm^−1^ (corresponding to the CH deformation of the β-glycosidic bond) and 1157 cm^−1^, corresponding to the saccharide structure [13,14]. The peaks in the range from 1515 to 1570 cm^−1^ were related to the amide II. Peaks corresponding to amide I and II were observed at 1650 and 1322 cm^−1^, respectively [13,14]. The peaks at 1595 and 1591 cm^−1^ indicated the deacetylation of the chitosan. The peaks in the region between the 1300 and 1200 cm^−1^ were related to the vibrations of NHCO. The peaks between 1000 and 1158 cm^−1^ were related to the vibrational modes of the C–O–C, C–OH and C–C bonds in the ring of the saccharide structure [13]. The skeletal vibrations of the pyranose ring related to NH and OH out of the plane were observed at 600 cm^−1^ [14,45].

The pristine Gel IR spectrum (Figure 3) showed the following most representative peaks: 1625 cm^−1^ (C=O stretching/hydrogen bonding couple with COO), 1500 cm^−1^ (amide II: N–H bend coupled with C–N stretch), 1435 cm^−1^ (amide II: CH_2_ bend), 1250 cm^−1^ (amide III: N–H bend), 1100 cm^−1^ (amide III: C–O stretch) and 1000–950 cm^−1^ (amide III: skeletal stretch) [51,52]. The IR spectrum of pristine PVA (Figure 3) showed a strong peak at 1085 cm^−1^ (C–O stretching and O–H bending, and the amorphous sequence of PVA), medium intensity at 1400 cm^−1^ (CH_2_ bend), 1350 cm^−1^ (scissoring O–H, rocking with C–H, wagging), 915 cm^−1^ (CH_2_ rocking) and 820 cm^−1^ (C–C stretching) [53].

The FTIR spectrum of the hydrogel exhibited the characteristic bands of the CS, Gel, and PVA. The dominant component of the hydrogel was identified mainly by the amide I (at 1630–1600 cm^−1^), amide II (at 1540–1350 cm^−1^) and amide III (at 1250–1300 cm^−1^) bands present in CS and Gel. The peaks correlated to the saccharide structure (1200–890 cm^−1^) of CS, and prominent peaks of the PVA correlated to the backbone of the PVA structure (1400–1300 cm^−1^). The amorphous region (1100–1080 cm^−1^), the CH_2_ asymmetric stretching (920 cm^−1^) and the skeletal vibration of PVA (830 cm^−1^) were also present.

However, some changes were noticeable in the CS/Gel/PVA hydrogel spectrum, as detailed below:

(1) The characteristic peaks of amide I and II exhibited displacements due to hydrogen bonding interactions between the C=O groups from the gelatin and the OH groups in the PVA and CS. Sánchez Cardona et al. (2021) found similar results, indicating that the three polymers interact through hydrogen bonding between the amino and hydroxyl moieties [23]. According to Rodríguez-Rodríguez et al. (2019), steam sterilization induces the molecular interaction between the amino groups of the CS and Gel and between either primary hydroxyl groups or the carbonyl under high temperatures [21].

(2) The displacement band corresponding to the C=O (1650–1550 cm^−1^) in CS and Gel suggested interactions between the functional groups of CS and the carboxyl groups in Gel, in agreement with Chang et al. (2009) [21]. Moreover, the peak at ~1550 cm^−1^ diminished in the hydrogel spectra, probably due to the formation of –NH_3_^+^ in the acidic environment and the transition to –NH_2_ under alkaline conditions [14]. The samples were immersed in NaOH to favor physical gelation throughout the deprotonation of the amino groups of CS (pKa = 6.3–7.0) [21]. The peak at ~1650–1640 cm^−1^ was related to the C=N bond, characteristic of the Schiff’s base structure, assuming that a covalent bond formation was maintaining the 3D structure of the hydrogel. Wang et al. (2004) demonstrated the formation of Schiff’s base and NH_3+_ in a CS–PVA hydrogel [14]. Corona-Escalera et al. (2022) suggested the formation of covalent bonds by an esterification reaction between free carboxylic and hydroxyl groups in Gel/PVA hydrogels crosslinked with transglutaminase enzyme [54].

(3) Alcohol gradient solutions were used to remove traces of o-xylene. The absence of the characteristic bands of the o-xylene at 743 cm^−1^ in the spectra highlighted the success of the o-xylene extraction process [55]. The effectiveness of xylene as a porogenic template has been demonstrated in CS [55] and CS/Gel/PVA hydrogels [21]. It is well-known that xylene dissolves alcohol during tissue processing in histology [56].

(4) The disappearance of the PVA characteristic peaks at 920 cm^−1^ and 830 cm^−1^ was noticeable, suggesting bonding interactions between CS/Gel and PVA molecules [57]. The vibration of aliphatic ether was associated with the stretching vibration, favoring a physical crosslinking of polymer chains with a hydroxyl group (such as CH_2_–CHO–CH_2~_) that interacted with other hydroxyl groups of PVA polymer chains, forming bindings such as C–O–C by removing small molecules, such as water molecules [57], due to the xylene/alcohol treatment, since the PVA showed an affinity with o-xylene [58].

**Table 1:** Main IR peak assignments for the chitosan (CS), gelatin (Gel), and poly(vinyl alcohol) (PVA).

Polymer	Peak Region (Wavenumber cm^−1^)	Assignment	Reference
Chitosan	~1655–1649	Amide I: C=O stretch	[14]
	~1650–1580	NH_2_ deformation	[14]
	~1570–1510 (1554)	Amide II: N–H deformation and C–N stretching	[14]
	~1625–1500 1560, 1548	NH_3_^+^ deformation Symmetric deformation	[14]
	~1420	O–H and C–H deformation (ring)	[14]
	~1383–1377	CH_3_ deformation (bend)	[14]
	~1325–1322	Amide III: O–H and C–H deformation	[14]
	~1265–1260	C–H wag (ring)	[14]
	~ 1200–950	C–O–C and C–O stretching	[13]
	~1160–1150	Primary or secondary alcohol	[14]
	~1080	C–O stretching	[14]
	~900	Aliphatic aldehydes/	[14]
	860–650	CH deformation of the β-glycosidic bond	[13]
Type B gelatin from bovine skin	~ 1650–1630	Amide I: C=O and N–H stretching /hydrogen bonding coupled with C00-	[51,52]
	~1632	Intermolecular associations with imide residues	[59]
	~1560–1540	Amide II: N–H bend coupled with C–N stretch	[51]
	~1460–1430	Amide II: CH_2_ bend	[51]
	~1250–1180	Amide III: N–H bend	[51]
	~1130–1022	Amide III: C–O stretch	[51]
	~1200–950	Amide III: skeletal stretch	[51]
	~890–650	Amide III: skeletal stretch	[51]
PVA	~1750–1735	C=O vibration	[53]
	~1640	Water absorption	[60]
	~1460–1400	CH_2_ bend	[53]
	~1350–1300	Scissoring O–H, rocking with C–H, wagging	[53]
	~1140–1080	C–O (crystallinity)	[53]
		C–O stretching of acetyl group present in the PVA backbone and O–H bending/ the amorphous sequence of PVA	[53]
	~920	CH_2_ rocking	[53]
	~820	C–C stretching/skeletal vibration	[53]

#### 3.2.3. Amino Acid Profile

The amino acid content of pristine type B gelatin from bovine skin (Sigma-Aldrich) and CS/Gel/PVA hydrogel samples are given in Table 2. The amounts of different amino acids were expressed in grams of amino acid per 100 mg of gelatin sample (g/100 g) and hydrogel. Gelatin and hydrogel samples did not exhibit tryptophan or cysteine because they are typically absent in type I collagen. Gelatin is partially denatured collagen, so the gelatin amino acid composition remains very similar to collagen [61].

Collagen contains more glycine than most amino acids but does not contain cysteine or tryptophan (except for type III collagen) [62]. However, the dominant amino acid in pristine bovine gelatin was Proline (Pro, 16.24%), followed by Glycine (Gly, 14.40%) and Histidine (His, 10.38%), which are characteristic of all gelatins [63]. Pro is the amino acid with the highest content during hydrogel preparation. Pro is an amino acid responsible for the stability of the triple helix in the collagen structure [63,64]. The contents of Alanine (Ala, 9.07%), Threonine (Thr, 4.59%), and Isoleucine (Ile 3.09%) were higher than the other amino acids present in the pristine bovine gelatin. Aykin-Dinçer et al. (2017) reported the amino acid profile of different commercial bovine gelatins, with differences in the amino acid profile and contents [63], which may be associated with the sources of skin and the gelatin manufacturing processes [64].

It is notorious that the amino acid content was lower in the hydrogels than in pristine gelatin (Pro (0.82%), Gly (0.73%), His (0.45%), Ala (0.39%), Thr (0.33%) and Ile (0.20%), suggesting chemical interactions between gelatin and the components of the hydrogel during the preparation process. The functional groups of gelatin peptides are involved in H-bonding and NH-bonding formation between the water molecules and the components of the hydrogel (CS and PVA), as corroborated by the FTIR analyses [23,45].

### 3.3. Culture of Chondrocytes in Chitosan/Gelatin/Poly(Vinyl Alcohol) Hydrogel

The performance of chondrocytes embedded in hydrogels was evaluated by measuring cell cytotoxicity, viability and attachment, and the formation of ECM proteins.

#### 3.3.1. Cytotoxicity Assay

**Figure 4:**
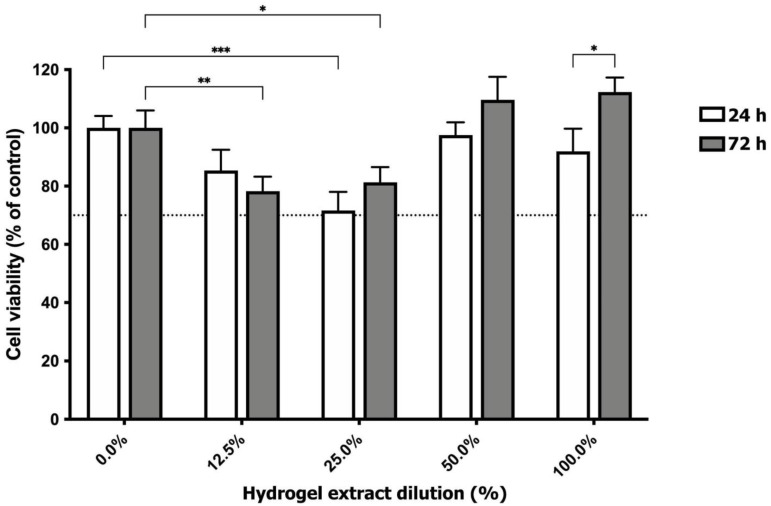
Cell viability of human auricular chondrocytes exposed to CS/Gel/PVA hydrogel extract dilutions. MTT assay was performed to evaluate cellular cytotoxicity 7 and 24 h post-exposure to extract dilutions. Notably, only the treatments marked with asterisks exhibited statistically significant differences from the control group. Therefore, 50 and 100% extract dilutions did not demonstrate statistically significant effects. Data are presented as the mean ± SD of l% of cell viability (n = 3) of three independent experiments. Statistical significances for each condition vs. non-exposed cells, or between conditions, are indicated by * *p* < 0.05, ** *p* < 0.01, and *** *p* < 0.001 (two-way ANOVA and Bonferroni’s multiple comparisons test).

Cytotoxicity is one of the most important properties of biomaterials for tissue engineering applications. To evaluate the cytocompatibility of the steam-sterilized hydrogels, isolated auricular chondrocytes were cultured on the different extracts after 24 and 72 h of incubation, and cell viability was measured using the MTT assay. Figure 4 presents the cell viability in the presence of CS/Gel/PVA hydrogel extracts. All samples showed a cell viability greater than 70%, regardless of the extract concentration. According to ISO10993-5 (ISO10993-5, 1999), the extract medium obtained from the sterilized hydrogels does not contain toxic substances to auricular chondrocyte cells, indicating good cytocompatibility for subsequent assays [21,23,41,44].

However, lower extract concentrations (12.5% and 25%) exhibited a statistically significant decrease in chondrocyte viability compared to the control group (non-exposed cells) at 72 and 24 h incubation, respectively. Conversely, no significant differences were observed at either 24 or 72 h for the highest tested concentrations (50.0% and 100%), compared to non-exposed cells. Interestingly, chondrocytes cultured in 50% and 100% extract concentrations demonstrated the highest viability after 72 h, with a statistically significant increase observed in the 100% extract group between 24 and 72 h. This could be explained by the presence of a higher amount of Gel dissolved in the culture medium than in the other extract dilutions, promoting cell growth and proliferation due to similarities in structure and function with collagen, which is an excellent substrate for cell attachment, proliferation and differentiation [23,65,66].

Previous studies have documented the cytocompatibility of sterilized CS/Gel/PVA hydrogels. Rodríguez-Rodríguez et al. (2020) reported no observable changes in the viability of HT29 cells cultured on extracts prepared via freeze–thawing and autoclaving (the same preparation method but a different batch than those employed in the present study) [21]. Pérez-Díaz et al. (2023) confirmed the biocompatibility of autoclaved 2D CS/Gel/PVA hydrogels by observing the favorable viability of human adipose tissue-derived mesenchymal stromal cells [20]. Massarelli et al. (2021) evaluated the cytocompatibility of NIH/3T3 fibroblasts on autoclaved CS/PVA hydrogels for potential application as a wound dressing [67]. Lastly, Sánchez-Cardona et al. (2021) reported a cell viability greater than 50% of rat pancreatic beta cells (BRIN-BD11) in the presence of CS/Gel/PVA hydrogels that were prepared in a similar way to those used in this study but without NaOH/Xylene/alcohol washes and autoclave sterilization [23].

#### 3.3.2. Cell Viability Assay

**Figure 5:**
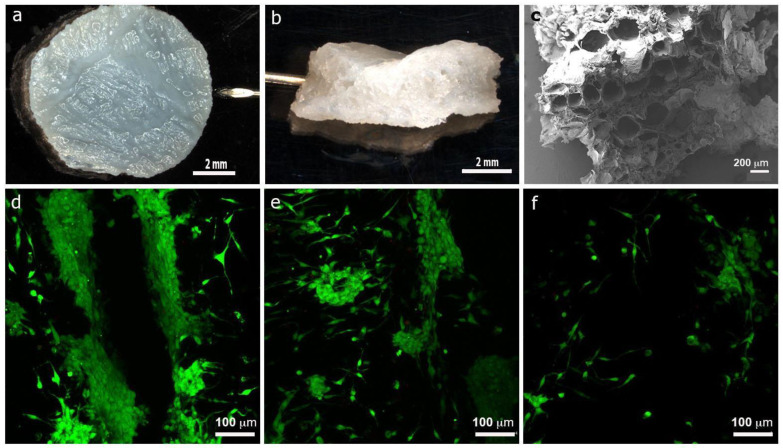
Cell-laden CS/Gel/PVA based hydrogel. Auricular chondrocytes (5 × 10^5^ cells) were injected into eight mm diameter CS/Gel/PVA hydrogel discs (**a**) and a Live/Dead viability test was performed and visualized via confocal microscopy. Hydrogels side view (**b**). SEM micrograph shows porous microstructure (**c**). Viable auricular chondrocytes were visualized near the injection site (**d**) and diffused all through the hydrogel network (**e**,**f**). Scale bars: 2 mm (**a**,**b**), 200 μm (**c**) and 100 μm (**d**–**f**).

Cell viability was investigated by live/dead staining after chondrocytes were encapsulated in the CS/Gel/PVA hydrogels and cultured for 7 and 14 days (Figure 5).

The strategy followed for chondrocyte encapsulation was injecting the cell suspension into the hydrogel. It was crucial to know if the structure in the wet state continued to maintain its porous interconnected structure and pore sizes in this condition. The macroscopic appearances of the sterilized hydrogels in a wet state are shown. The hydrogels showed a sponge-like structure with smooth surface topography. The hydrogels showed optimal pore size ranges (greater than 200 μm) and an irregular macrostructure that favors the exchange of nutrients and gases and, therefore, the growth and functionality of chondrocytes (Figure 5a–c).

Auricular chondrocytes embedded in the CS/Gel/PVA construct were identified, confirming that the injected cell suspension favored cell diffusion and distribution into the hydrogel. In all groups, most of the chondrocytes were alive (green signal), and dead cells were observed (red signal) after 7 and 14 days of culture (Figure 5d–f, respectively). The results indicated that the cells in the hydrogels had high cell viability, suggesting that using the CS/Gel/PVA hydrogels for cell encapsulation may be a good strategy. An increased number of viable chondrocytes were found near the injection site, while chondrocyte density diminished in areas far from the injection site (Figure 5d–f).

**Figure 6:**
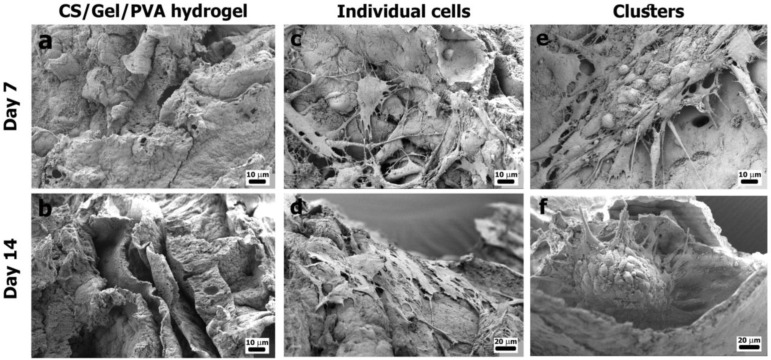
Cell attachment onto CS/Gel/PVA hydrogels. Auricular chondrocytes were cultured onto CS/Gel/PVA hydrogels in standard culture conditions. SEM microscopy was used to visualize cell attachment. Hydrogel without cells after 7 (**a**) and 14 days’ (**b**) culture. Individual (**c**,**d**) and clustered cells growth (**e**,**f**) after seven and 14 days, respectively. Scale bars 10 μm (**a**–**c**,**e**) and 20 μm (**d**,**f**).

Confocal images revealed cell proliferation in cell-laden hydrogels with two notable growth patterns: individual cells and aggregated cells (clusters). Similar cell behavior was observed when auricular chondrocytes were encapsulated in fibrin–agarose hydrogels mimicking a 3D environment [68]. For cells that showed individual growth, polygonal morphology was also observed, and the viability was 98.8% by day seven and 98.69% after 14 days of culture (Figure 6a,b). In contrast, clusters showed 99.21% viability by day seven and 98.96% after 14 days of culture, corroborated by the live/dead staining. However, viability between different conditions was not statistically different. Furthermore, the hydrogel stiffness and smooth surface did not influence cell viability. Rodriguez-Rodriguez et al. (2020) demonstrated that sterilized CS/Gel/PVA hydrogels showed higher elastic modulus values than a viscous modulus, indicating that the hydrogels are soft and have predominantly solid viscoelastic behavior [21].

Auricular cartilage comprises chondrocytes and a highly hydrated extracellular matrix, characterized by chondrocytes immersed in an ECM rich in elastic and type II collagen fibers, as well as proteoglycans [69]. The 3D organization of ECM molecules confers elasticity, flexibility, tensile resistance and compression to the elastic cartilage [70]. Several biomaterial scaffold fabrication techniques have been established to process different microstructures with controlled characteristics that mimic the chondrocytes’ ECM [71,72]. Characteristics such as porosity, pore size and interconnectivity must be adequate for cells to carry out their primary functions and transport and exchange nutrients to chondrocytes [37]. Lien et al. (2019) demonstrated that a pore size between 250 and 500 μm was optimal for articular chondrocytes to proliferate, to form ECMs and to maintain their phenotype using scaffolds [73].

#### 3.3.3. Cell Attachment

The cell attachment of auricular chondrocytes was evaluated by SEM (Figure 6). The micrographs show CS/Gel/PVA hydrogels without cells in culture conditions after 7 and 14 days (Figure 6a,b). Cell attachment and ECM deposition in cell-laden CS/Gel/PVA-based hydrogels also confirmed the presence of individual cells and clusters observed in Live/Dead staining. Cells remained attached throughout the hydrogel surface. Remarkably, after 14 days of culture, chondrocytes showed more cell growth and ECM deposition than after 7 days. Individual cells showed prolonged filopodia in contact with nearby cells (Figure 6c,d). Clusters possessed a round, nodule-like shape with lamellipodia and filopodia projected towards the periphery of the conglomerates (Figure 6d,e). Results reported by Otto et al. (2018) demonstrated the in vitro cartilage-forming capacity of primary auricular chondrocytes seeded onto gelatin methacryloyl (gelMA)-based hydrogels after 56 days of culture. Chondrocytes displayed a more cluster-like organization of ECM components in a 3D microenvironment, with cartilage-specific matrix deposition and increased ECM production [38].

Initially, the manual cell seeding technique at a high density resulted in the formation of a dense layer condensed in the area where the cell suspension was first seeded. However, later, we determined that cell injection, combined with a 3D interconnected network and the macroporous structure of the hydrogel, provided a better environment for the growth and functionality of chondrocytes [39]. It was found that the surface chemistry provided by the composition of the CS/Gel/PVA polymer, macroporous structure, and stiffness, was favorable for the culture of these particular cells. Also, it is well known that clustered chondrocytes promote differentiation into elastic chondrocytes. Clusters are aggregates where chondrogenesis and osteogenesis take place, in order to form cartilage and bones during embryonic development or repair/regeneration [74,75].

#### 3.3.4. Elastic Cartilage Extracellular Matrix Formation

**Figure 7:**
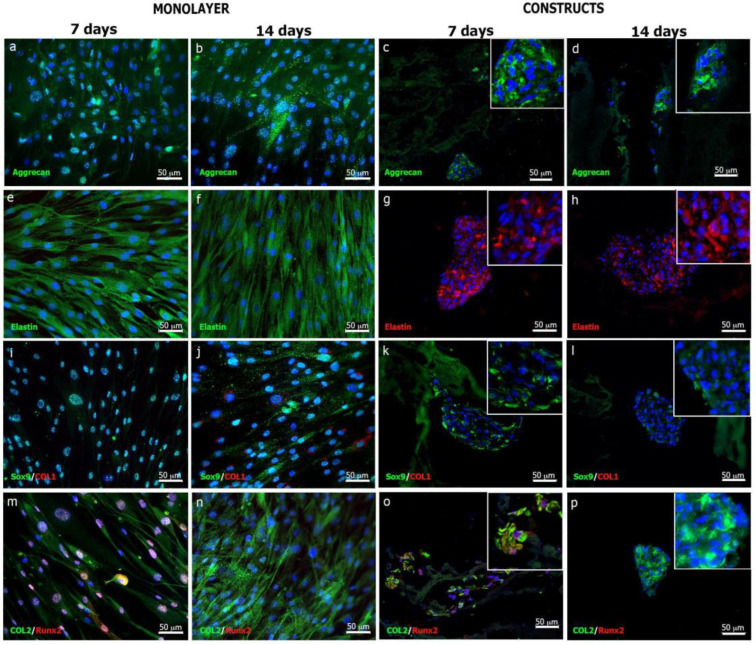
Immunostaining of cartilage markers. Third passage elastic cartilage chondrocytes were cultured in monolayers and onto 3D CS/Gel/PVA hydrogels (constructs) for 7 and 14 days (**d**), respectively. Aggrecan (ACAN) expression was consistent in monolayer (**a**,**b**) and in constructs (**c**,**d**) after 7 and 14 d. Elastin was also expressed in monolayer cells ((**e**,**f**), green signal) and constructs ((**g**,**h**), red signal) at both time frames. Expression of chondrogenic factor SOX9 was detected after 7 and 14 d in monolayer (**i**,**j**) and 7 d constructs (**k**); however, expression diminished in 14 d constructs (**l**). For the osteogenic marker, type I collagen (COL1) expression in monolayer was absent at 7 d (**i**) with a slight increase by 14 d (**j**); however, expression was not detected during 7 or 14 d culture onto 3D CS/Gel/PVA hydrogels (**k**,**l**). Positive expression of type II collagen (COL2), a cartilage ECM marker, was detected in chondrocytes on both monolayer (**m**,**n**) and constructs (**o**,**p**) after 7 and 14 d. Osteogenic factor Runx2 was expressed during the initial stages of monolayer (**m**) or construct (**o**) culture; however, expression was not detected after 14 d culture onto 3D CS/Gel/PVA hydrogels (**n**,**p**). The expression of chondrogenic and osteogenic markers was analyzed using LSM 880 Zeiss confocal microscope. Scale bars are 50 μm.

The expression of elastic cartilage ECM proteins by chondrocytes on monolayer and onto CS/Gel/PVA hydrogels was investigated using immunofluorescence (Figure 7). After in vitro expansion, auricular chondrocytes cultured in monolayer expressed aggrecan (ACAN) a widely distributed proteoglycan in articular hyaline cartilage, elastic cartilage and fibrocartilage (Figure 7a,b), [76]. The expression of elastin (Figure 7e,f), the most abundant fibrous protein in elastic cartilage ECM [48], was also detected. The expression of Sox9, the intrinsic transcription factor related to determining and maintaining chondrogenic lineage, was confirmed. After 14 days in culture, the remaining expression of Sox9 was detected in the nuclei of chondrocytes (Figure 7j); however, COL1 expression was absent at seven days (Figure 7m), and slightly present at 14 days in monolayer culture (Figure 7i,j). However. the expression of type II collagen (COL2), the main collagen in elastic cartilage, was detected by day 7 and significantly increased by day 14 (Figure 7n) Ref [77]. Also, the slight expression of the transcription factor RUNX2 was detected at 7 days (Figure 7m). COL1 and RUNX2 are markers of osteoblast differentiation and cartilage maturation, respectively. Meanwhile, the RUNX2 expression stimulates lineage differentiation toward osteoblasts and contributes to chondrocyte development by regulating maturation into hypertrophic chondrocytes [78]. The expression of these markers demonstrated that auricular chondrocytes from patients with auricular microtia did not lose their phenotype and characteristics as elastic chondrocytes in monolayer culture.

The expression of ECM proteins in 3D CS/Gel/PVA hydrogels showed differences compared to monolayer cultures. Chondrocytes that were densely arranged/distributed around cartilaginous clusters expressed higher levels of ACAN and elastin after 7 and 14 days of culture (Figure 7c,d,g,h). This confirmed the expression of elastic cartilage-specific ECM molecules in the 3D microenvironment. Although the expression of Sox9 was evident by day 7, it decreased by day 14 (Figure 7k,l). Noticeably, COL2 expression increased from day 7 to day 14 (Figure 7o,p). Moreover, Runx2 expression was not detected on CS/Gel/PVA hydrogels, while COL1 had a slight but not relevant expression from day 7 to day 14 (Figure 7k,l).

To our understanding, the preservation of a 3D microenvironment is key to promoting and maintaining chondrogenic phenotypes and the expression of cartilage markers such as elastin, aggrecan and type II collagen [29,30,38,68,79]. The expression of these markers demonstrated that auricular chondrocytes from microtia patients did not lose their phenotype, either in monolayer cultures or after being cultured onto CS/Gel/PVA-based hydrogels. Therefore, these hydrogels can be used as 3D cell supports for potential auricular cartilage regeneration.

## 4. Conclusions

The CS/Gel/PVA hydrogel showed promising potential for cartilage tissue regeneration, providing the basis for microtia reconstruction based on the culture of auricular chondrocytes from microtia in a 3D environment. The manufacturing process of the CS/Gel/PVA hydrogels is feasible, robust and easily reproducible, providing a reliable method to produce hydrogels with customized shapes and maintaining a pattern in the pore formation, providing a structurally stable, 3D, macroporous and highly interconnected network. The CS/Gel/PVA hydrogels can maintain the phenotype of auricular chondrocytes in culture, avoiding the process of cellular dedifferentiation in fibrocartilage cells usually found in monolayer cultures. Furthermore, long-term cultures of the chondrocyte hydrogel construct revealed a favorable environment that maintains the intrinsic cellular characteristics of elastic cartilage by maintaining its chondral phenotype, cellular cluster formation and elastic cartilage ECM production. These results are an important step toward building bioengineered auricular cartilage tissue.

## Figures and Tables

**Table 2:** Amino acid composition of commercial type B gelatin from bovine skin (Sigma-Aldrich) and CS/Gel/PVA hydrogel.

Amino Acid	Pristine Type B Gelatin from Bovine Skin% (g/100 g)	CS/Gel/PVA Hydrogel % (g/100 g)
Histidine (His)	10.38	0.45
Serine (Ser)	1.13	0.08
Arginine (Arg)	0.29	0.01
Glycine (Gly)	14.40	0.73
Aspartic acid (Asp)	ND	ND
Glutamic acid (Glx)	0.98	0.03
Threonine (Thr)	4.59	0.33
Alanine (Ala)	9.07	0.39
Proline (Pro)	16.24	0.82
Lysine (Lys)	0.01	0.01
Tyrosine (Tyr)	ND	ND
Valine (Val)	0.63	0.08
Isoleucine (Ile)	3.09	0.20
Leucine (Leu)	1.54	0.01
Phenylalanine (Phe)	0.01	0.02

Note: ND: not detected.

## Data Availability

The data presented in this study are available on request from the corresponding author. The data are not publicly available due to intellectual property reasons.
